# A new tibial insert design with ball-in-socket medial conformity and posterior cruciate ligament retention has low tibial baseplate migration after unrestricted kinematically aligned total knee arthroplasty: a cohort study using radiostereometric analysis

**DOI:** 10.2340/17453674.2024.42489

**Published:** 2024-12-23

**Authors:** Abigail E NIESEN, Pranav A TIRUMALAI, Stephen M HOWELL, Maury L HULL

**Affiliations:** 1 Department of Biomedical Engineering, University of California, Davis, CA; 2 Department of Mechanical Engineering, University of California, Davis, CA; 3 Department of Orthopaedic Surgery, University of California Davis Medical Center, Sacramento, CA, USA

## Abstract

**Background and purpose:**

In total knee arthroplasty (TKA), an insert with ball-in-socket (BS) medial conformity (MC) and posterior cruciate ligament (PCL) retention restores kinematics closer to native than an insert with intermediate (I) MC. However, high medial conformity might compromise baseplate stability as indicated by maximum total point motion (MTPM). Using the BS MC insert with PCL retention, we aimed to determine whether (i) the baseplate is stable as indicated by mean MTPM < 0.5 mm, (ii) baseplate stability is not strongly correlated to varus baseplate alignment, and (iii) baseplate stability, clinical outcome scores, and flexion are comparable with that of an I MC insert cohort which has demonstrated high stability, clinical outcome scores, and flexion.

**Methods:**

Unrestricted kinematic alignment (unKA) TKA was performed on a cohort of 35 patients using a cemented baseplate. Biplanar radiographs acquired at timepoints up to 12 months were processed with model-based radiostereometric analysis (RSA) software to determine MTPM.

**Results:**

At 1 year, mean MTPM of 0.35 mm was significantly below 0.5 mm (P < 0.001). MTPM was not strongly correlated to varus baseplate alignment up to 9° (r = 0.12, 95% confidence interval –0.22 to 0.44). Equivalence analyses revealed that MTPM, Forgotten Joint Score, Oxford Knee Score, and maximum flexion for the sBS MC insert were comparable with the I MC insert.

**Conclusion:**

Using the new BS MC insert with PCL retention, the tibial baseplate was stable at the group level at 1 year. Baseplate stability was not strongly related to varus baseplate and limb alignment. Comparable patient-reported outcome scores and maximum flexion/extension at 1 year were shown between the 2 insert designs.

Unrestricted kinematically aligned (unKA) total knee arthroplasty (TKA) generally restores tibiofemoral joint kinematic axes and hence function close to native when used in conjunction with an insert that offers a high degree of medial conformity (MC), has a flat lateral articular surface, and retains the PCL. However, a concern is the possible loss of fixation, resulting in a high incidence of tibial baseplate loosening due to varus alignment. This concern stems from the result that tibial baseplates malaligned in greater than 3º of varus in mechanical alignment (MA) have increased migration [1,2] and a concomitant relatively high incidence of aseptic loosening [3,4].

Although a new tibial insert design with ball-in-socket (BS) MC restores tibiofemoral kinematics more closely to native than an insert with intermediate (I) (i.e., less than spherical) MC [5,6] (Figure 1), the new BS MC insert, which maximizes A–P stability, exacerbates concerns regarding baseplate loosening due to the potential for increased A–P shear loads. This is because the medial femoral condyle in the healthy knee displaces in the A–P direction in gait [7]. Hence, the added constraint provided by BS conformity might reasonably be expected to increase A–P shear loads, which could cause aseptic loosening. Accordingly, assessing the risk of long-term baseplate loosening of this new BS MC design when used in unKA is of high interest. Also of interest is whether increased varus alignment is associated with an increased risk of baseplate loosening. If not associated, then there would be no evidence to support restricting varus alignment of the tibial baseplate as imposed by restricted KA for example [8].

**Figure 1:**
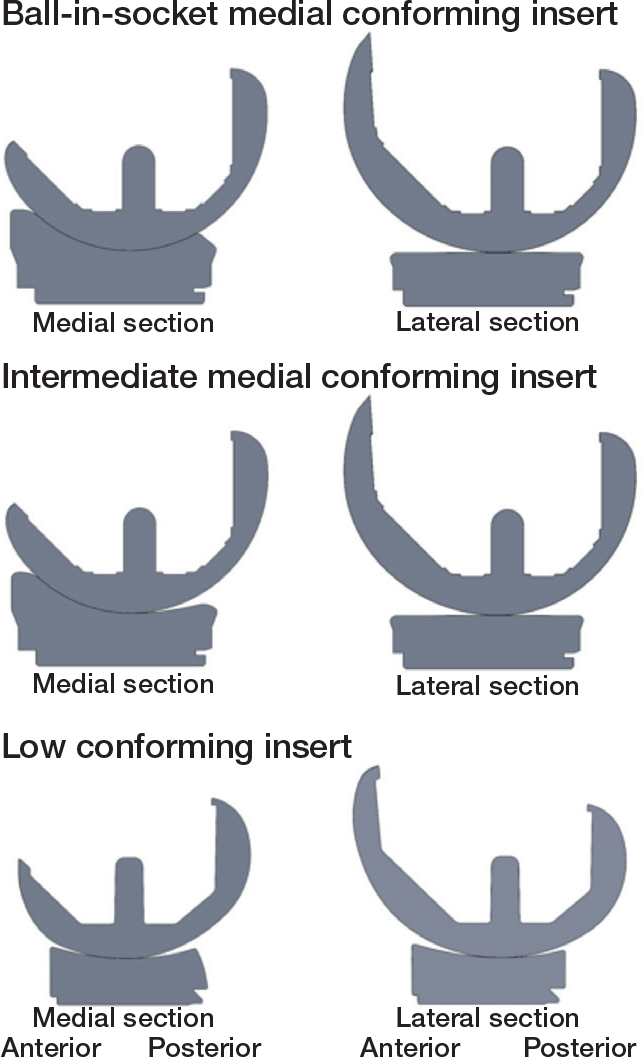
Section views of the femoral condyles and tibial insert showing articular geometry in the medial and lateral compartments for 3 insert designs. The medial conforming designs are Size 4 GMK Sphere (Medacta, Castel San Pietro, Switzerland) and were used in the present study. The BS MC insert and the previously studied I MC insert have the same flat lateral articular surface, but different profiles in the medial compartment. For the BS MC insert, the profile through the center of the medial condyle is a true ball-in-socket design. For the previously studied I MC insert, the profile through the center of the medial condyle has a true ball-in-socket anterior half and a less than ball-in-socket posterior half, which are separated by a 3 mm flattened surface (i.e., large radius of curvature). Both inserts articulate with a medial femoral condyle that has a single radius from –5° to 115° flexion. The example low conforming insert design is a cruciate-retaining Size 7 Persona (Zimmer-Biomet, Warsaw, IN, USA).

As with any new implant design, assessing knee function following unKA TKA is of interest. Because of higher conformity posteriorly (Figure 1), the new BS MC insert may reduce range of motion, hence reducing clinical outcome scores [9,10] compared with the lower conforming I MC insert, in which case the I MC insert would be preferrable to patients.

Using radiostereometry analysis (RSA), our primary aim was to determine whether the tibial baseplate with the BS MC insert is stable as indicated by mean maximum total point motion (MTPM) less than 0.5 mm at 1 year [11]. Secondary aims were to determine whether baseplate stability is not strongly correlated to varus baseplate and limb alignment, and whether baseplate stability at 1 year, clinical outcome scores, and flexion with the BS MC are comparable to a previously studied I MC insert which has demonstrated high stability, function, and flexion [12].

## Methods

### Patients

Inclusion criteria were patients with symptomatic osteoarthritis of the knee aged 40–85 years, who were able to give informed consent, who did not have a prior joint implant in the treated limb, who did not have a history of neurological disorder, who did not have a history of rheumatoid arthritis, who were not pregnant, and who were not currently involved in healthcare litigation. No restriction was placed on the severity of preoperative varus–valgus or flexion-contracture deformity. For the BS MC insert, all patients were enrolled at 1 site between August 2021 and February 2022 (Table 1). Patient enrollment information for the 35 patients included in the I MC insert cohort has been published previously [12]. The study was reported according to STROBE guidelines.

**Table 1:** Patient demographics and clinical characteristics. Values are median (interquartile range) unless otherwise specified

	BS MC insert	I MC insert	P value
Demographics			
Age, mean (SD)	67 (6)	68 (7)	0.4
Male sex, n (%)	19 (54)	21 (60)	0.8
Body mass index, mean (SD)	31 (6)	31 (5)	0.7
Preoperative motion and deformity			
Flexion contracture (°)	10 (0–13)	6 (4–10)	0.4
Flexion (°)	118 (111–124)	115 (108–120)	0.07
Varus (+)/valgus (–) deformity (°)	10 (6–13)	9 (–8 to 12)	0.2
Kellgren–Lawrence, n grade 3 / grade 4	12/23	11/24	1.0
Preoperative function scores			
Oxford Knee score **^a^**	22 (13–29)	25 (17–29)	0.3
1-year postoperative motion			
Extension (°)	0 (0–2)	1 (0–2)	
Flexion (°)	118 (115–125)	120 (110–125)	
1-year postoperative function scores			
Forgotten Joint score **^b^**	73 (44–90)	73 (48–88)	
Oxford Knee score **^a^**	44 (40–46)	44 (38–46)	

a 48 best, 0 worst

b 100 best, 0 worst

SD = standard deviation.

### Surgical technique

A single experienced surgeon (i.e., > 5,000 unKA TKAs) performed caliper-verified, unrestricted KA TKA using manual instruments. Caliper-verified unKA TKA is a patient-specific approach in which resections are made to restore the patient’s pre-arthritic joint lines and limb alignment without collateral ligament release. The accuracy with which this is accomplished using manual instruments has been described previously [12]. Patients received a cemented femoral component with a spherical femoral condyles, a cemented anatomic tibial baseplate with the stem cemented, a fixed-bearing BS MC tibial insert (Figure 1) with a flat lateral articular surface and PCL retention, and a cemented anatomic patella component (GMK Sphere, Medacta, Castel San Pietro, Switzerland). PCL sufficiency was checked by palpation when put under tension using a laminar spreader after the resections were made, by palpation with trial components sans tibial insert in place, and by a posterior drawer test with the knee at 90º flexion. Insert thickness, which maximized internal tibial rotation between 0° and 90° flexion, was determined using an insert goniometer [13]. Before cementing the baseplate, 8 to 10 tantalum beads of 1.0 mm diameter were inserted in the cancellous bone of the proximal tibia (Halifax Bead Set and Inserter; Halifax Biomedical Inc., Nova Scotia, Canada) to create a bone reference for measuring migration. Details of baseplate cementation were described previously [12].

### Postoperative limb and implant alignment

Postoperatively on the day of surgery, non-weightbearing, anteroposterior, rotationally controlled, long-leg CT scanograms were acquired and used to measure the medial proximal tibial angle (MPTA) and hip–knee–ankle angle (HKAA) (Table 2). Reliability of measurements for MPTA and HKAA were previously evaluated where intraclass correlation (ICC) values for inter-observer and intra-observer variability were 0.76 to 0.97, indicating good to excellent agreement, and repeatability was 0.3° and 0.2° for MPTA and HKAA, respectively [12].

**Table 2:** Postoperative radiographic alignment measurements. Values are mean (standard deviation) [range]

Alignment	BS MC insert	I MC insert	P value
MPTA (°) **^a^**	4 (2.1) [–0.2 to 8.7]	6 (2.1) [0.9 to 9.7]	0.002
HKAA (°) **^b^**	1 (3.6) [–7.1 to 7.2]	2 (2.8) [–4.9 to 7.8]	0.085

a MPTA = Medial proximal tibial angle of implant, varus (+)/valgus (–).

b HKAA = Hip-knee-ankle angle of limb, varus (+)/valgus (–).

### Biplanar imaging and migration analysis

The image acquisition and analysis have been described previously for the I MC insert [12]. Briefly, biplanar radiographs were acquired on the day of surgery (i.e., baseline examination) using 2 portable X-ray machines (HF80H+; MinXray, Northbrook, IL, USA) mounted 90° with respect to one another and oriented perpendicular to a calibration box (Model 10; Tilly Medical Products AB, Lund, Sweden). The patient’s knee was centered in the calibration box and flexed slightly to project the tibial baseplate oblique to the image plane, which improved accuracy when registering the 3D baseplate model to the image silhouette [14]. To assess baseplate migration, additional biplanar radiographs were obtained at follow-up examinations of 1.5 months, 3 months, 6 months, and 1 year. Radiographs were processed using model-based RSA software (RSAcore, version 4.2, Leiden, The Netherlands) using reverse-engineered (RE) 3D baseplate models, which have lower registration error than CAD models. Migration was the difference in the position and orientation of the RE model at the follow-up examinations relative to the baseline examination.

**Figure 2:**
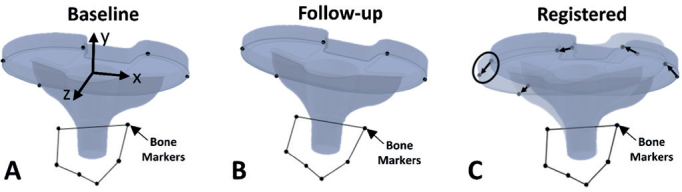
Images demonstrating the tibial baseplate coordinate system, location of the 5 feature points on the baseplate, and process for determining maximum total point motion (MTPM). Positive coordinate system directions for a right-sided baseplate were x = medial, y = proximal, and z = anterior and rotations were computed using an xyz Cardan angle sequence. The 5 feature points were the most anterior point, the most medial point, the most posterior point on the medial side of the baseplate, the most posterior point on the lateral side of the baseplate, and the most lateral point. To determine MTPM, the 5 baseplate feature points and bone markers were located on (A) the images at the baseline examination and (B) the images at each follow-up examination. Next, the bone markers were registered, and the 5 baseplate feature points were connected with vectors (C). MTPM was the magnitude of the largest vector (circled).

Migration was computed in 6 degrees of freedom consistent with ISO16087 [15] (Table 3, see Appendix). Because the baseplate was not oriented parallel to the walls of the calibration box, the migration computation was based on a local baseplate coordinate system (Figure 2) [16,17]. The coordinate system origin was the point where the central axis of the baseplate stem intersected the lower surface of the baseplate tray. MTPM was computed using 5 standardized feature points on the bottom surface of the baseplate (Figure 2). The maximum condition number and mean error of rigid body fitting were 80 and 0.26 mm, respectively, which are below recommended values of 120 and 0.35 mm [15].

**Table 3:** Tibial baseplate migration in 6 degrees of freedom and in MTPM at 4 follow-up timepoints relative to the baseline examination. Values are mean (standard deviation)

Follow-up	Implant	n	Translations (mm)			Rotations (°)			MTPM (mm)
			medial (+) lateral (–) (x)	proximal (+) distal (–) (y)	anterior (+) posterior (–) (z)	flexion (+) extension (–) (x)	internal (+)external (–) (y)	valgus (+) varus (–) (z)	
1.5 months	BS MC	35	0.01 (0.15)	0.01 (0.06)	0.02 (0.08)	0.00 (0.12)	0.00 (0.25)	–0.01 (0.18)	0.28 (0.15)
	I MC	34	0.04 (0.14)	0.00 (0.05)	0.05 (0.11)	0.05 (0.18)	0.05 (0.31)	–0.04 (0.17)	0.31 (0.19)
3 months	BS MC	35	–0.02 (0.14)	0.01 (0.06)	0.00 (0.09)	–0.03 (0.12)	–0.04 (0.24)	0.03 (0.17)	0.26 (0.17)
	I MC	35	0.05 (0.14)	0.00 (0.04)	0.06 (0.13)	0.07 (0.20)	0.06 (0.29)	–0.04 (0.15)	0.30 (0.18)
6 months	BS MC	35	–0.03 (0.14)	0.01 (0.07)	0.02 (0.12)	–0.02 (0.16)	–0.05 (0.31)	0.04 (0.18)	0.29 (0.19)
	I MC	34	0.03 (0.14)	0.01 (0.05)	0.05 (0.13)	0.02 (0.18)	–0.03 (0.24)	–0.03 (0.17)	0.29 (0.16)
1 year	BS MC	35	–0.05 (0.17)	0.02 (0.08)	0.03 (0.15)	–0.04 (0.20)	–0.04 (0.30)	0.06 (0.23)	0.35 (0.21)
	I MC	34	0.01 (0.17)	0.02 (0.05)	0.07 (0.17)	0.04 (0.22)	0.01 (0.28)	0.01 (0.21)	0.35 (0.21)

Mean measurement error (bias) and standard deviation of error (repeatability) of baseplate migration were quantified based on 2 independent examinations termed double examinations [15] on the same day. Mean measurement error was the mean migration between the 2 sets of bi-planar radiographs from the double examinations (Table 4) [17,18]. Repeatability was the standard deviation of the difference in migration between each set of radiographs from the double examinations referenced to the baseline examination (Table 4) [15].

**Table 4:** Mean measurement error and repeatability of tibial baseplate migration in 6 degrees of freedom and in MTPM based on double examinations

Factor	Implant	n	Translations (mm)			Rotations (°)			MTPM (mm)
			medial (+) lateral (–) (x)	proximal (+) distal (–) (y)	anterior (+) posterior (–) (z)	flexion (+) extension (–) (x)	internal (+)external (–) (y)	valgus (+) varus (–) (z)	
Measurement error, mean	BS MC	35	0.00	0.00	–0.02	–0.02	–0.01	0.01	0.18
	I MC	35	0.02	0.02	–0.01	–0.02	–0.03	–0.02	0.21
Repeatability, SD	BS MC	35	0.07	0.03	0.06	0.09	0.21	0.08	0.11
	I MC	35	0.08	0.03	0.05	0.08	0.23	0.09	0.13

### Statistics

To determine the sample size, a power analysis based on a one-sample t-test was performed for the primary aim. A single-sided analysis was used because the aim was to determine whether mean MTPM was less than the 0.5 mm stability limit. 27 patients were required to detect a difference in MTPM of 0.1 mm using 0.2 mm standard deviation in MTPM [19], α = 0.05, and β = 0.80. To account for 20% dropout, the sample size was 35 patients.

To satisfy the 1st aim, a one-sample t-test was used to determine whether mean MTPM at 1 year was significantly less than 0.5 mm for the BS MC insert. To satisfy the 2nd aim, Pearson’s correlation coefficient together with 95% confidence interval (CI) were computed to assess how strongly MPTA and the HKAA were associated with MTPM for the BS MC insert at 1 year. To satisfy the 3rd and final aim, Wilcoxon equivalence analyses were performed for each dependent variable because dependent variables exhibited skewed distributions. Differences to detect were 0.1 mm for mean MTPM, 14 points for the Forgotten Joint Score (FJS) [20], 5 points for the Oxford Knee Score (OKS) [21], 10° for maximum flexion [22], and 5° for extension [23]. In all statistical tests, α = 0.05, in which case the equivalence analyses were performed at 90% confidence (i.e., (1–2α)x100%).

### Ethics, registration, data sharing, use of AI, funding, and disclosures

The Institutional Review Board of the University of California, Davis approved the study (IRB No. 1251807-10). The authors received financial support from Medacta USA, Inc. Complete disclosure of interest forms according to ICMJE are available on the article page, doi: 10.2340/17453674.2024.42489

## Results

**Figure 3:**
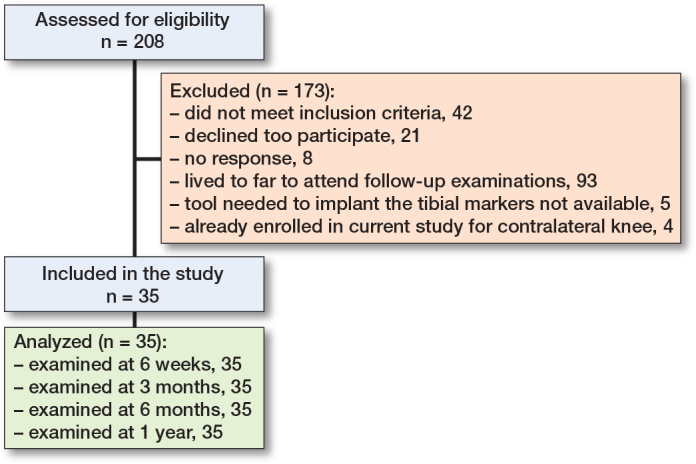
Patient flow diagram.

208 patients were considered for eligibility during the period of enrollment. Of these, 173 were excluded for the main reasons that they lived more than 1 hour from the hospital (n = 93), did not meet the inclusion criteria (n = 42), or declined to participate (n = 21). Hence, 35 patients provided informed consent and participated (Figure 3).

**Figure 4:**
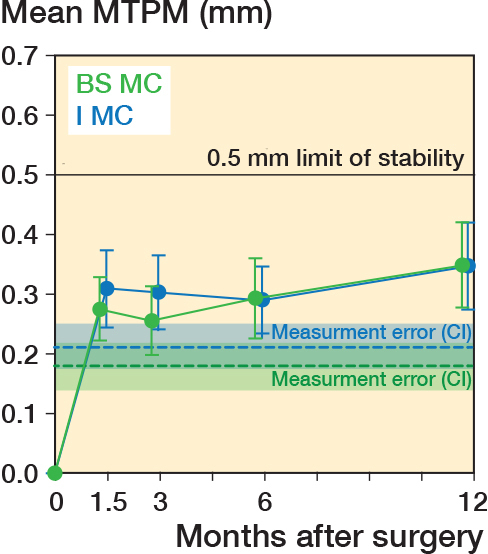
Plot of mean maximum total point motion (MTPM) of the tibial baseplate for the BS MC insert (green) and I MC insert (dark blue) over 1 year. Error bars represent the 95% confidence intervals on the means. Mean measurement error (dashed lines) and 95% confidence limits (shaded area) determined from double examinations are shown.

**Figure 5:**
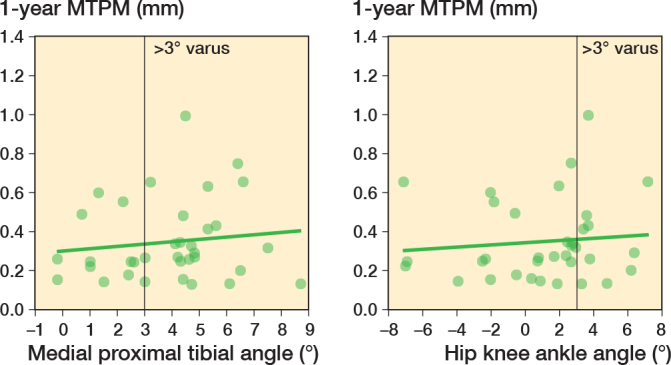
Postoperative medial proximal tibial angle (left panel) and hip–knee–ankle angle (right panel) vs maximum total point motion (MTPM) of the tibial baseplate at 1 year for 35 patients with the BS MC insert.

Regarding the first 2 aims, at 1 year mean MTPM of 0.35 mm using the BS MC insert was significantly less than 0.5 mm (P < 0.001; Figure 4). The correlation analysis confirmed that MTPM was not strongly correlated to varus alignment of the baseplate up to 9° (r = 0.12, CI –0.22 to 0.44; Figure 5) and varus alignment of the limb up to 8° (r = 0.10; CI –0.24 to 0.42, Figure 5).

Comparing the new BS MC insert with the previously studied I MC insert using the equivalence analyses, none of the differences to detect were included in the corresponding confidence intervals. The 0.1 mm difference to detect for MTPM was not included in the confidence interval (CI –0.06 to 0.07 mm, P = 0.008), the 14-point difference to detect in the FJS was not included in the equivalence interval (EI –10.4 to 10.4), the 5-point difference to detect in the OKS was not included in the equivalence interval (EI –2.0 to 1.0), the 10° difference to detect in maximum flexion was not included in the equivalence interval (EI –5° to 3°) and the 5° difference to detect in extension was not included in the equivalence interval (EI 0.0°–1.0°).

## Discussion

Using the new BS MC insert in unrestricted KA, we showed that (1) the tibial baseplate was stable, (2) baseplate stability was not strongly associated with varus baseplate and limb alignment, and (3) baseplate stability, patient-reported outcome scores, flexion, and extension for the BS MC insert were comparable to the I MC insert, which has demonstrated high stability, patient-reported outcome scores, and flexion [12].

To assess risk of baseplate loosening at the group level, MTPM was computed and the mean at 1 year was compared with a 0.5 mm stability limit determined from a meta-analysis [11]. One important finding was that the mean MTPM of 0.35 mm was significantly below the 0.5 mm stability limit at 1 year, indicating a low risk of baseplate loosening for unKA TKA using a cemented, fixed-bearing, BS MC insert, which maximizes A–P stability. This finding holds notwithstanding that our implant alignment and design might very well be a worst case given the varus baseplate alignment, the maximum A–P constraint of the medial femoral condyle afforded by ball-in-socket conformity of the insert, and unconstrained posterior movement of the lateral femoral condyle afforded by the flat lateral surface of the insert. The latter could lead to posterior subsidence as a result of repeated posterolateral edge loading. Despite being a worst case, our results are similar to those reported for cemented, unrestricted KA and a low-conforming tibial insert, which had a mean MTPM of 0.41 mm at 1 year [24]. In contrast to the insert used herein, the low-conforming insert design allows A–P movement of the medial femoral condyle and blocks posterior movement of the lateral femoral condyle [25].

Although our mean MTPM of 0.35 mm was significantly below the 0.5 mm stability limit at 1 year, another study using the same implant design—albeit with the PCL sacrificed—reported a significantly greater mean of 1.0 mm [26]. This increase was due in part to registration error and its effect in increasing mean MTPM. To minimize registration errors in our study, reverse-engineering models were used, and imaging planes were oblique to cardinal body planes, which showed more geometric details of the baseplate. Using CAD models instead of reverse-engineering models increased mean MTPM by 0.07 mm [27], whereas using imaging planes consistent with cardinal body planes increased mean MTPM by up to 0.3 mm [14].

As registration errors do not fully account for the increase, other factors should be considered. For example, the well-documented biomechanical shortcomings of mechanical alignment (MA) compared with unKA could also in part explain the increase. When tibial compartment forces are averaged at 0°, 45°, and 90° of flexion, MA forces are 3–4 times and 5–6 times higher in the medial and lateral compartments, respectively, and imbalance is 1.5–3 times higher than unKA even after ligament release in MA [28]. Moreover, MA creates a postoperative joint line oblique to the ground, increases the knee adduction moment [29], and does not reproduce native kinematics during gait as well as KA [30].

A second important finding was that baseplate stability with the BS MC insert was not strongly related to baseplate and limb varus alignment, despite 69% of patients having a MPTA > 3° varus and 31% of patients having a HKAA > 3° varus. In fact, given that the confidence interval on the correlation coefficients included 0, it is possible that MTPM is strictly unrelated to varus alignment of the baseplate and of the limb. A similar finding has been previously reported for the I MC insert where the percentage of patients having an MPTA and HKAA > 3º was even higher [12]. This result further confirms that restricting alignment to within ± 3° for KA is unnecessary as stability at 1 year is unaffected by varus baseplate and limb alignment.

Final important findings were that baseplate stability as indicated by MTPM, clinical outcome scores, and range of motion were comparable for the 2 inserts. Starting with MTPM, a difference of less than 0.1 mm at 1 year indicates that surgeons who prefer to retain the PCL and use unKA can transition from an I MC to a BS MC insert without concern regarding an increased risk of baseplate loosening.

Next, considering outcome scores, restoration of function is an important goal of TKA where an FJS of 80 and 66 indicate an artificial joint with no restriction and minimal restriction, respectively [31]. Median 1-year FJSs of 73 and OKSs of 44 for the BS MC and I MC inserts indicate that high function was achieved. Because patient-reported outcomes scores of the 2 inserts were comparable, neither insert offers an advantage over the other in superior function.

Finally, although the PCL is often resected using an insert with BS medial conformity, our study shows that flexion was not compromised. As flexion was not compromised and because retaining the PCL in unKA offers the benefits of improved posterior stability [32], increased internal tibial rotation in flexion comparable to native [33], and preservation of the native joint lines [33], the PCL should be preserved in unKA TKA.

### Limitations

Limitations using the I MC insert have been previously described in detail [12] and the same limitations apply to the present comparison study as well. Namely, our results apply to cemented baseplates using unKA TKA and do not reflect migration of uncemented baseplates. Second, 1 implant design was evaluated whereas other implant designs may experience different migration magnitudes. Third, 1 experienced surgeon performed the unKA TKA procedure. Although risk of loosening may differ for other surgeons, this is unlikely because caliper-verified unKA TKA uses intraoperative verification checks to ensure alignment targets are met and recent studies demonstrate that surgeon experience has a negligible effect on accuracy of the femoral resections [34]. Finally, this study evaluated migration using unKA performed with manual instruments in which the accuracy of the caliper-verified technique has been evaluated thoroughly. Results may not apply to other types of instrumentation, which achieve different accuracy. However, use of other types of instrumentation is unnecessary in performing unKA TKA because alignment targets are achieved consistently and efficiently with manual instruments.

An additional limitation is that ideally the present study would have been a randomized controlled trial (RCT) rather than a cohort comparison. However, the 2 cohorts of patients were recruited from the same geographic area within a relatively short time period of about 1 year. Although the 2 cohorts exhibited no significant differences in demographics, nevertheless unknown confounders could still be present. A significant postoperative difference between the 2 cohorts was that patients with the I MC insert had significantly more varus mean MPTA than patients with the BS MC insert, which we do not believe is clinically relevant. Next, the patients who participated in the present study were not a consecutive series and only about 18% of patients who had surgery during the enrollment period participated, which could have introduced sampling bias. As noted earlier, the most common reason for exclusion was that the patient lived too far away, which was unlikely to introduce bias.

Finally, the 0.5 mm stability limit is derived from a meta-analysis of 53 RSA studies in which cohorts with a 1-year mean MTPM below 0.5 mm had a risk of baseplate loosening of less than 5% at 10 years [11]. As these 53 studies used MA TKA, caution should be exercised in applying the stability limit to unKA TKA considering the systematic differences in baseplate alignment between unKA and MA.

### Conclusion

Using the new BS MC insert with PCL retention, the tibial baseplate was stable at the group level at 1 year. Baseplate stability was not strongly related to varus baseplate and limb alignment. Comparable patient-reported outcome scores and maximum flexion/extension at 1 year were shown between the 2 insert designs.

## Notes

AEN coordinated imaging of patients, imaged patients, analyzed images, performed statistical analyses, and wrote the draft of the manuscript; PAT assisted with image analysis; SMH performed unKA TKA on patients and provided supervision; MLH conceived the study, obtained funding, supervised all aspects, edited the manuscript, and revised the manuscript.

The authors are grateful to Medacta USA, Inc. for financial support.

Handling co-editors: Marianne Westberg abd Philippe Wagner

*Acta* thanks Rob Nelissen and Stephan Maximilian Röhrl for help with peer review of this manuscript.
